# Population structure analysis to explore genetic diversity and geographical distribution characteristics of cultivated-type tea plant in Guizhou Plateau

**DOI:** 10.1186/s12870-022-03438-7

**Published:** 2022-01-27

**Authors:** Zhifei Zhao, Qinfei Song, Dingchen Bai, Suzhen Niu, Yingqin He, Dahe Qiao, Zhengwu Chen, Caiyun Li, Jing Luo, Fang Li

**Affiliations:** 1 College of Tea Science / Tea Engineering Technology Research Center, Guizhou University, Guiyang, 550025 Guizhou Province PR China; 2 lnstitute of Tea Science, Guizhou Academy of Agricultural Sciences, Guiyang, 550006 Guizhou Province PR China

**Keywords:** Cultivated-type tea plant, Genetic diversity, Genotyping-by-sequencing, Guizhou Plateau, Population structure

## Abstract

**Background:**

Tea plants originated in southwestern China. Guizhou Plateau is an original center of tea plants, and is rich in germplasm resources. However, the genetic diversity, population structure and distribution characteristics of cultivated-type tea plants in the region are unknown. In this study, we explored the genetic diversity and geographical distribution of cultivated-type tea accessions in Guizhou Plateau.

**Results:**

We used 112,072 high-quality genotyping-by-sequencing to analyze the genetic diversity, principal components, phylogeny, population structure, and linkage disequilibrium, and develop a core collection of 253 cultivated-type tea plant accessions from Guizhou Plateau. The results showed Genetic diversity of the cultivated-type tea accessions of the Pearl River Basin was significantly higher than that of the cultivated-type tea accessions of the Yangtze River Basin. Three inferred pure groups (CG-1, CG-2 and CG-3) and one inferred admixture group (CG-4), were identified by a population structure analysis, and verified by principal component and phylogenetic analyses. The highest genetic distance and differentiation coefficients were determined for CG-2 vs CG-3. The lower genetic distance and differentiation coefficients were determined for CG-4 vs CG-2 and CG-4 vs CG-3, respectively. We developed a core set and a primary set. The primary and core sets contained 77.0 and 33.6% of all individuals in the initial set, respectively. The primary set may serve as the primary population in genome-wide association studies, while the core collection may serve as the core population in multiple treatment setting studies.

**Conclusions:**

The present study demonstrated the genetic diversity and geographical distribution characteristics of cultivated-type tea plants in Guizhou Plateau. Significant differences in genetic diversity and evolutionary direction were detected between the ancient landraces of the Pearl River Basin and the those of the Yangtze River Basin. Major rivers and ancient hubs were largely responsible for the genetic exchange between the Pearl River Basin and the Yangtze River Basin ancient landraces as well as the formation of the ancient hubs evolutionary group. Genetic diversity, population structure and core collection elucidated by this study will facilitate further genetic studies, germplasm protection, and breeding of tea plants.

**Supplementary Information:**

The online version contains supplementary material available at 10.1186/s12870-022-03438-7.

## Background

Tea (*Camellia sinensis*) is one of the three most widely consumed beverages worldwide. It provides numerous cultural, health and economic benefits [1]. Tea extracts are rich in secondary metabolites [2], including polyphenol, theanine, caffeine, polysaccharides and volatile oils. These substances are antioxidant, stimulant, diuretic, hypoglycemic and immunomodulatory [2–6]. Tea plants originated in southwestern China, and are cultivated in over 60 and spread to over 160 countries. This crop has a significant impact on the agricultural economy [1, 7]. Whereas the spread and cultivation of tea has flourished in the past, future challenges of the global tea industry include low breeding efficiency and the lack of excellent varieties [8]. However, germplasms are the invaluable fundamental resources for genetic crop improvement, determine the success of breeding programs, and have attracted widespread research attention [9].

The Pearl River Basin (PR Basin) and the Yangtze River Basin (YR Basin) are vital water sources basin in southern China and are especially suitable for tea plant growth [10–12]. Previous studies showed that several ancient tea plant varieties are distributed in the YR Basin and its southern reaches in Guizhou, Yunnan, and Guangxi Provinces. Higher levels of genetic diversity have been detected in the tea plants of these regions [1, 8, 13]. To protect the environment of the middle and lower reaches of the YR and PR Basins, economic and land use have been methodically and gradually developed in the upper reaches of the region. Consequently, there has been no large-scale elimination of various tea varieties [12, 14, 15]. Guizhou Plateau is one of the original centers of tea plant and is located in the upper reaches of the YR and PR Basins. Assuming Miaoling Mountain as the dividing line, the southern part of Guizhou Plateau is located in the PR Basin, while its northern part is located in the YR Basin [16, 17]. Guizhou Plateau has abundant tea germplasms with high genetic diversity and various morphological characteristics, such as modern landraces, ancient landraces and wild germplasms because of slow land use and economic development, tea plant self-incompatibility and allogamy, and a long history of cultivation [9, 15, 18, 19]. However, exploitation of these resources has been limited, they had complex genetic backgrounds, and their degree of domestication was unclear. Research of the genetic diversity of tea germplasms has expanded our knowledge of the origins and population structures of tea plants. This information will facilitate the breeding of improved varieties and development of tea industry [8]. An earlier study explored the genetic diversity of tea plants by using various molecular markers, such as RAPD [20], SSR [21], EST-SSR [22, 23], and AFLP [24]. The advent of next-generation sequencing technologies has led to the application of genotyping-by-sequencing (GBS) which is a rapid, cost-effective utility for genotyping breeding populations [25], GBS has enabled plant breeders to implement genome-wide association studies (GWAS), genomic selection, and genomic diversity, and genetic linkage analysis, and to discover molecular marker in large-scale plant breeding programs [26]. GBS has been applied to wheat [27], maize [28], pepper [29], pine [30, 31] and tea [32]. A previous study reported that 79,016 high-quality single nucleotide polymorphisms (SNPs) were identified. A subsequent analysis revealed that both cultivated-type and wild-type tea plants were distributed on both sides of UPGMA tree, However, genetic diversity was higher for cultivated-type than wild-type tea plants. We selected 253 cultivated-type tea plants identified in prior research on tea plants and analyzed them in this study [15].

Earlier studies revealed that wind, water, animals, and human activity have contributed to the distribution and genetic diversity of *Sophora*, *Cycas*, *Spartina*, and other plant taxa [33–37]. However, similar large-scale studies have seldom been conducted on cultivated-type tea plants. To explore the genetic diversity, and geographical distribution characteristics of cultivated-type plants in Guizhou Plateau, we sampled 253 cultivated-type tea accessions from 32 regions distributed in seven water systems of the PR and YR Basins of Guizhou Plateau. Base on GBS method, we investigated the genetic diversity, population structure, and linkage disequilibrium (LD) using SNP data of the 253 cultivated-type tea accessions. We then elucidated the contributions of water basins, ancient hubs, and major rivers to the genetic diversity and distribution characteristics of cultivated-type tea plants in Guizhou Plateau. Finally, we constructed the core collection of these tea plant accessions.

## Results

### Sequencing and variant discovery

**Fig. 1:**
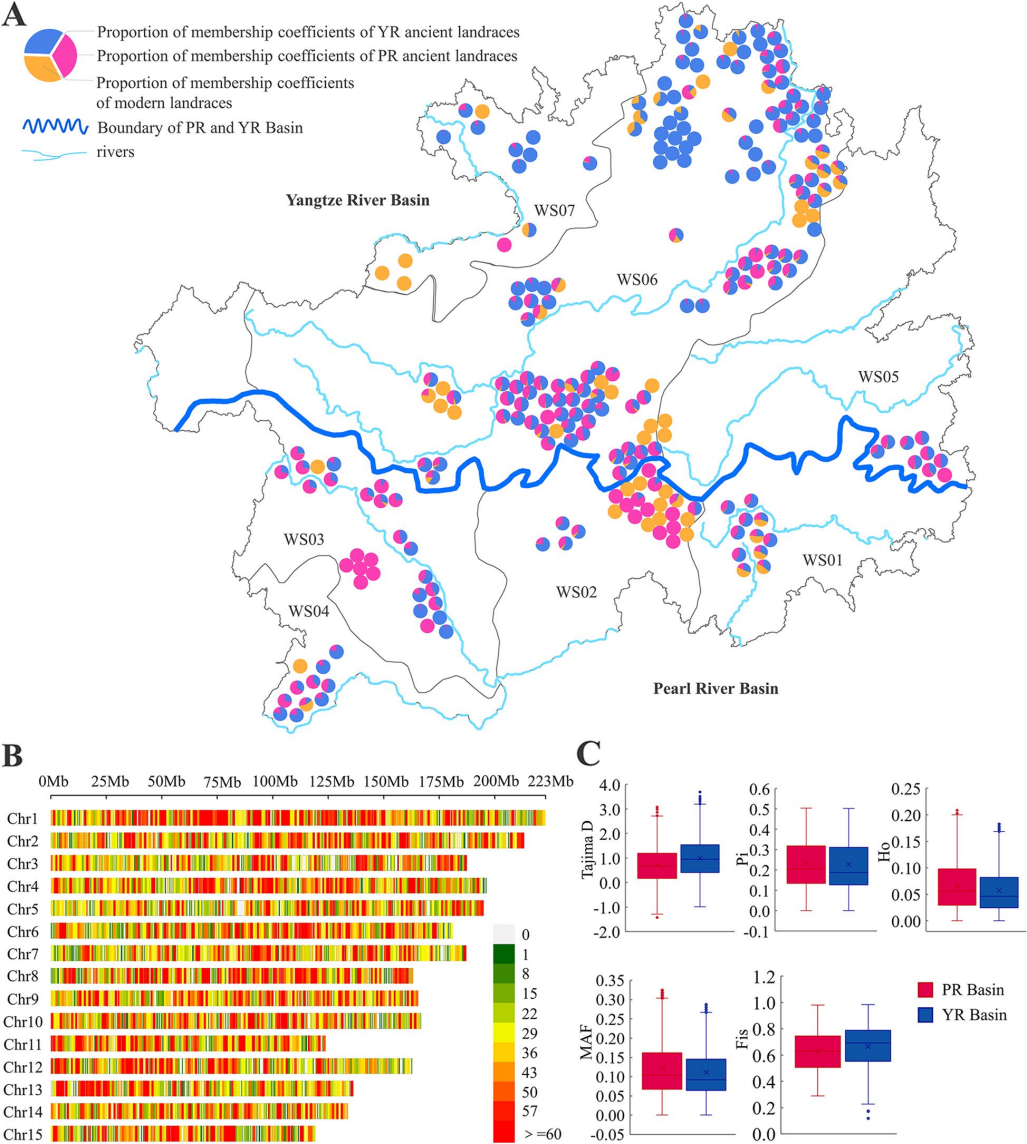
Geographic distribution, SNPs density and genetic diversity of 253 accessions. **A** Geographic distribution of each accession was represented by its’ pie chart of membership coefficient in ADMIXTURE on the Guizhou map [17]. For the three membership coefficients, CG-1 (modern landraces group) was in yellow, CG-2 (PR ancient landraces group) was in red and CG-3 (YR ancient landraces group) was in blue in the pie chart. **B** Distribution map of SNPs on 15 chromosomes graph [1]. **C** Comparison of the genetic diversity of both basins, PR Basin were showed in red and YR Basin were in blue. *Pi* nucleotide diversity, *Ho* observed heterozygosity, *MAF* minor allele frequency, *Fis* inbreeding coefficient

For this study, 253 cultivated-type tea plant accessions were used (Additional file 1: Table S1). Of these, 172 were ancient landraces and 81 were modern landraces. They were distributed in the PR and YR Basins of Guizhou Plateau. The geographical distribution of 249 accessions in the PR and YR Basins in Guizhou Plateau is shown in Fig. 1A. The other four accessions were introduced from other provinces and cultivated in tea gardens in Guizhou Plateau. A GBS analysis was performed on all 253 cultivated-type tea accessions using Illumina HiSeq Xten platform. We obtained 255.2 Gb clean data and an average of 1.00 Gb per accession (Additional file 2: Table S1). We mapped the clean reads to a tea reference genome sequence (http://tpia.teaplant.org/). GATK (v3.7.0) was used to detect and genotype the SNPs based on the reference genome [38]. We identified 29,393,327 SNPs. Filtering left 112,072 high-quality SNPs and the heterozygosity values were calculated. The average heterozygosity rate per accession was 7.89% (Additional file 2: Table S2). The SNPs were unevenly distributed over 15 chromosomes. The mean number of SNPs per chromosome was 6832. The lowest and highest SNP density were detected on chromosomes 5 and chromosomes 8, respectively (Fig. 1B). The nucleotide substitutions indicated that 112,072 SNPs were classified into transition and transversion. There were 1,585,601 (77.87%) transitions and 450,567 (22.13%) transversions. The substitution frequencies were 137,380 (6.75%) A/T, 112,861 (5.54%) A/C, 117,244 (5.76%) G/T, 83,082 (4.08%) C/G, 805,072 (39.54%) C/T, and 780,529 (38.33%) A/G. The transition to transversion ratio was 3.51 (Table 1).

**Table 1:** Percentage of transition and transversion SNPs identified using genotyping-by-sequencing

	Transitions		Transversions			
	CT	AG	AT	AC	CG	GT
Numbers of allelic sites	805,072	780,529	137,380	112,861	83,082	117,244
Percentage of allelic sites	39.54%	38.33%	6.75%	5.54%	4.08%	5.76%
Total (Percentage)	1,585,601 (77.87%)		450,567 (22.13%)			

### Genetic diversity estimation

Nucleotide diversity (*Pi*), observed heterozygosity (*Ho*), minor allele frequency (*MAF*) and inbreeding coefficient (*Fis*) were used as genetic diversity indicators. *Pi*, *Ho*, *MAF*, and *Fis* for the 253 cultivated-type tea accessions were 0.230, 0.082, 0.149 and 0.657, respectively (Table 2). We compared the genetic diversity of two tea plant populations in the PR and YR Basins of Guizhou Plateau. *Pi, Ho*, and *MAF* for the tea population in the PR Basin were significantly higher than those for the tea population in YR Basin (Table 2, Fig. 1C). In the PR Basin, *Pi*, *Ho* and *MAF* were significantly higher for the WS02 tea population than those in the other water systems. *Fis* was higher for the WS04 tea population than those in other water systems. In the YR Basin, *Pi* and *MAF* were significantly higher for the tea population in WS06 than for those in WS05 and WS07. *Fis* was higher for the tea population in WS07 than for those in the other water systems. We estimated the genetic diversity of the ancient landrace and modern landraces among the cultivated-type tea populations. *Pi* and *MAF* were significantly higher for the ancient than the modern landraces. *Ho* was significantly higher for the modern than the ancient landraces (Table 2).

**Table 2:** Genetic diversity parameters of 253 cultivated-type tea accessions in Guizhou Plateau

Type		Number	Tajima D	Pi	Ho	MAF	Fis
Basins	PR	75	0.752	0.234a	0.091a	0.151a	0.625a
	YR	174	1.048	0.228b	0.079b	0.148b	0.664a
Water systems	WS01	11	0.387	0.212f	0.103b	0.142d	0.460c
	WS02	26	0.380	0.232a	0.111a	0.151a	0.563bc
	WS03	27	0.321	0.225c	0.081d	0.147c	0.653ab
	WS04	11	0.337	0.218d	0.066e	0.142d	0.695a
	WS05	9	0.408	0.216e	0.083c	0.142d	0.612ab
	WS06	151	0.999	0.229b	0.081d	0.149b	0.658ab
	WS07	14	0.216	0.204 g	0.058f	0.133e	0.704a
Cultivation status	ML	81	0.593	0.218b	0.084a	0.142b	0.627a
	AL	172	1.098	0.232a	0.082b	0.151a	0.662a
All	all	253	1.236	0.230	0.082	0.149	0.657

Note: *Pi* nucleotide diversity, *Ho* observed heterozygosity, *MAF* minor allele frequency, *Fis* inbreeding coefficient; In the same type and line, the different letters indicate a significant difference in *p* = *0.05* levels by the T-test; *PR* Pearl River Basin contains *WS01* Liujiang River System, *WS02* Hongshui River System, *WS03* Beipanjiang River System and *WS04* Nanpanjiang River System. *YR* Yangtze River Basin contains *WS05* Yuanjiang River System, *WS06* Wujiang River System, *WS07* Chishui River System

Previous studies showed population bottlenecks and/or balancing selection when positive Tajima’s D values were determined for a population [39, 40]. The positive Tajima’s D values of all tea populations here suggest that they all underwent population bottlenecks and/or balancing selection (Table 2). Differentiation coefficients (*Fst*) is widely used as a measure of population structure, and the *Fst* in the range of 0.00–0.05 indicates little divergence while *Fst* in the range of 0.05–0.15 indicates moderate divergence [41–43]. *Fst* and genetic distance (*GD*) for the seven water systems showed that all pairwise *Fst* were < 0.05. Hence, there was little divergence among these populations. The highest pairwise *GD* were for WS04 vs WS02 and WS04 vs WS05. The lowest pairwise *GD* were determined for WS01 vs WS06 and for WS01 vs WS07 (Table 3).

**Table 3:** Pairwise *Fst* and GD among seven water systems of 253 accessions in Guizhou Plateau

	WS01	WS02	WS03	WS04	WS05	WS06	WS07
WS01		0.211	0.211	0.215	0.211	0.209	0.209
WS02	0.037b		0.220	0.225	0.224	0.221	0.219
WS03	0.034d	0.020 h		0.220	0.219	0.218	0.219
WS04	0.033d	0.019i	0.004 m		0.225	0.221	0.222
WS05	0.040a	0.027e	0.011 l	0.013 k		0.221	0.223
WS06	0.027e	0.021 g	0.011 l	0.002o	0.011 l		0.217
WS07	0.035c	0.016j	0.013 k	0.011 l	0.023f	0.003n	

Note: The bottom left is the value of pairwise genetic differentiation coefficients (*Fst*); The upper right is the value of pairwise genetic distance; The different letters indicate a significant difference in *p* = 0.05 levels by the T-test; WS01 Liujiang River System, WS02 Hongshui River System, WS03 Beipanjiang River System, WS04 Nanpanjiang River System, WS05 Yuanjiang River System, WS06 Wujiang River System, WS07 Chishui River System

### Population structure, PCA, and phylogenetic analysis

**Fig. 2:**
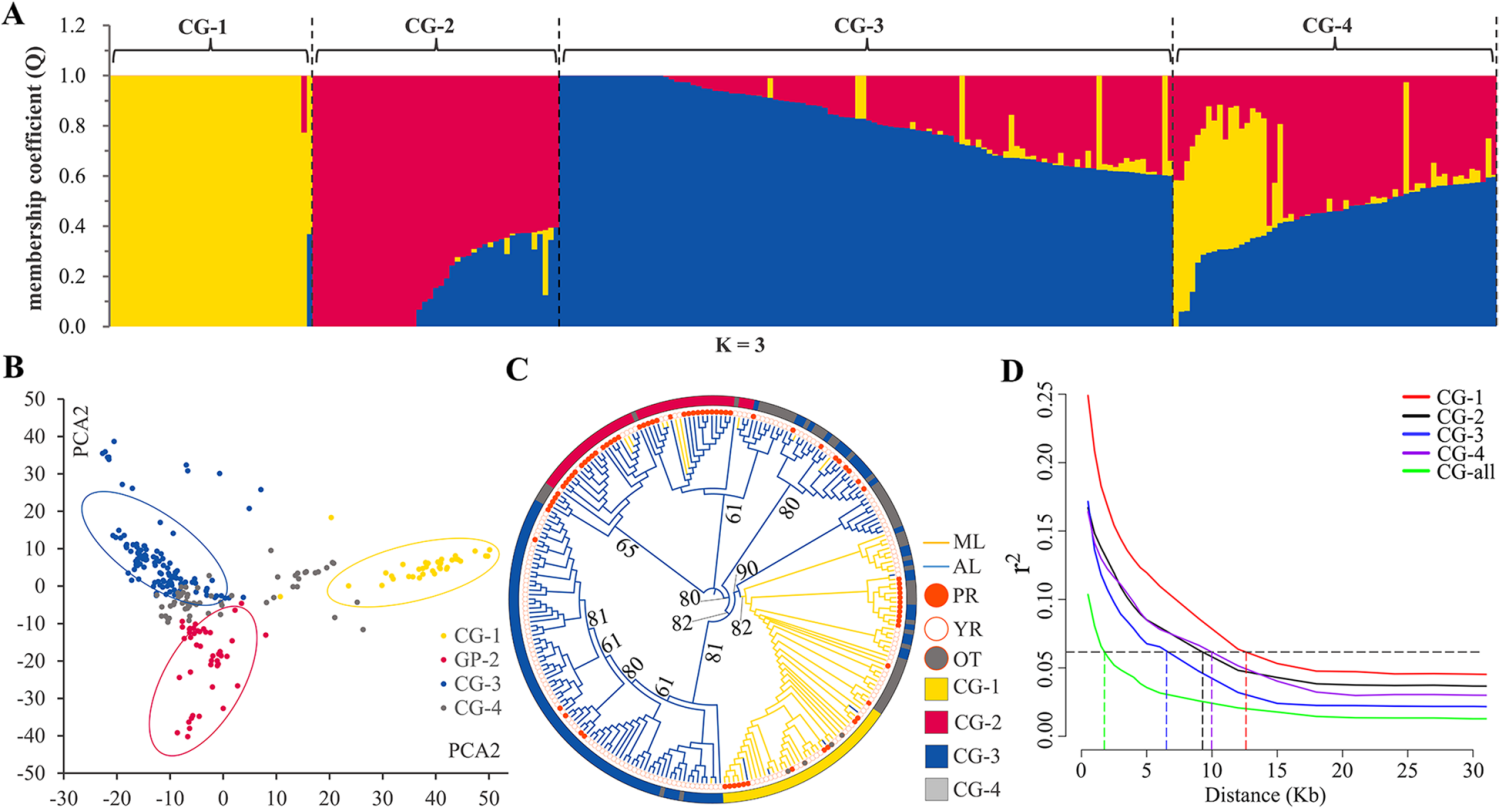
Population structure and LD of 253 accessions **A** Inferred population structure of 253 accessions. Bar plot of individual membership coefficients for the genetic clusters inferred using ADMIXITURE (K = 3) base on 112,070 SNPs. Individual membership coefficients (Q) were sorted within each cluster. CG-1, CG-2 and CG-3 are shown in yellow, red and blue, respectively. **B** Principal component analysis (PCA). The four PCA scatter diagram was made by the first and second principal components. Four inferred populations were identified in ADMIXITURE, CG-1 was showed in yellow, CG-2 in red, CG-3 in blue and CG-4 in grey. **C** NJ tree compared the classification of inferred populations, cultivated statuses (*ML* modern landraces, *AL* ancient landraces) and two basins (*PR* the Pearl River Basin, *YR* Yangtze River Basin and *OT* other). **D** The LD decay plot of 253 accessions and four inferred populations

We used 112,072 high-quality SNPs to analyze the population structure of the 253 cultivated-type tea plants and performed principal component analyses (PCA) on them. The cross-validation error (CV error) curve generated a minimum value when *k* equal 3. Thus, one admixture and three ancestral groups were identified (Fig. 2A, Additional file 3: Fig. S1). Accessions with membership coefficients > 0.60 were assigned to the corresponding pure groups. Those with coefficients < 0.60 were assigned to the admixture group (Additional file 1: Table S1) [44]. The first pure group contained 37 accessions, including 33 (89%) modern and 4 (11%) ancient landraces. The four introduced varieties were classified into this group (Additional file 1: Table S2). Henceforth, the designation is ‘modern landraces group’ or ‘CG-1’. The second pure group contained 45 accessions, including seven (16%) modern and 38 (84%) ancient landraces (Additional file 1: Table S2). Of the latter, 31 (82%) tea accessions were derived from the PR Basin and seven (18%) were derived from the YR Basin (Additional file 1: Table S2). Henceforth, the designation is ‘PR ancient landraces group’ or ‘CG-2’. The third pure group composed 112 accessions including 16 (14%) modern and 96 (86%) ancient landraces (Additional file 1: Table S2). Of the latter, 81 (84%) tea accessions were derived from the YR Basin and 15 (16%) were derived from the PR Basin (Additional file 1: Table S2). Henceforth, the designation is ‘YR ancient landraces group’ or ‘CG-3’. Fifty-nine tea accessions were assigned to the admixed group and included 25 modern and 34 ancient landraces (Additional file 1: Table S2). Fifty (84.7%) tea accessions were located near ancient hubs and nine (15.3%) were remote from them. The nine remote tea accessions consisted of seven accessions near major rivers and two accessions remote from them (Additional file 1: Table S3). Henceforth, the designation is ‘ancient hubs evolutionary group’ or ‘CG-4’. Of 59 tea accessions, thirteen were collected from the PR Basin and included six from WS01, three from WS02, two from WS03, and two from WS04. Another 46 accessions were collected from the YR Basin and included six from WS05, 39 from WS06, and one from WS07 (Additional file 1: Table S3).

The 112,072 SNPs of the 253 cultivated-type tea accessions were subjected to PCA and Neighbor-Joining tree (NJ tree) analysis to explore the cluster relationships and verify the stability of the potential population structure. The PCA and NJ tree disclosed four major clusters corresponding to CG-1, CG-2, CG-3, and CG-4. Hence, mutually verify the accuracy of population structure (Fig. 2B and C).

### LD analysis

LD analysis is used to clarify domestication and breeding history. We estimated LD for a population of 253 accessions by using 29,393,327 non-LD pruned SNPs. The LD rapidly decayed with increasing physical distance. The maximum r^2^ values were 0.12 for the LD decay of all 253 accessions. As r^2^ decayed to half maximum (0.06), the corresponding physical distance was ~ 2 kb (Fig. 2D).

The slowest LD was determined for CG-1. Its LD decay (r^2^ = 0.06) corresponded to a physical distance of ~ 13 kb. The physical distances for CG-4 and CG-2 were ~ 10 kb and ~ 9 kb, respectively. The fastest LD decay was determined for CG-3. The corresponding physical distance was ~ 6 kb (Fig. 2D).

### Genetic differentiation analysis of the inferred populations

**Fig. 3:**
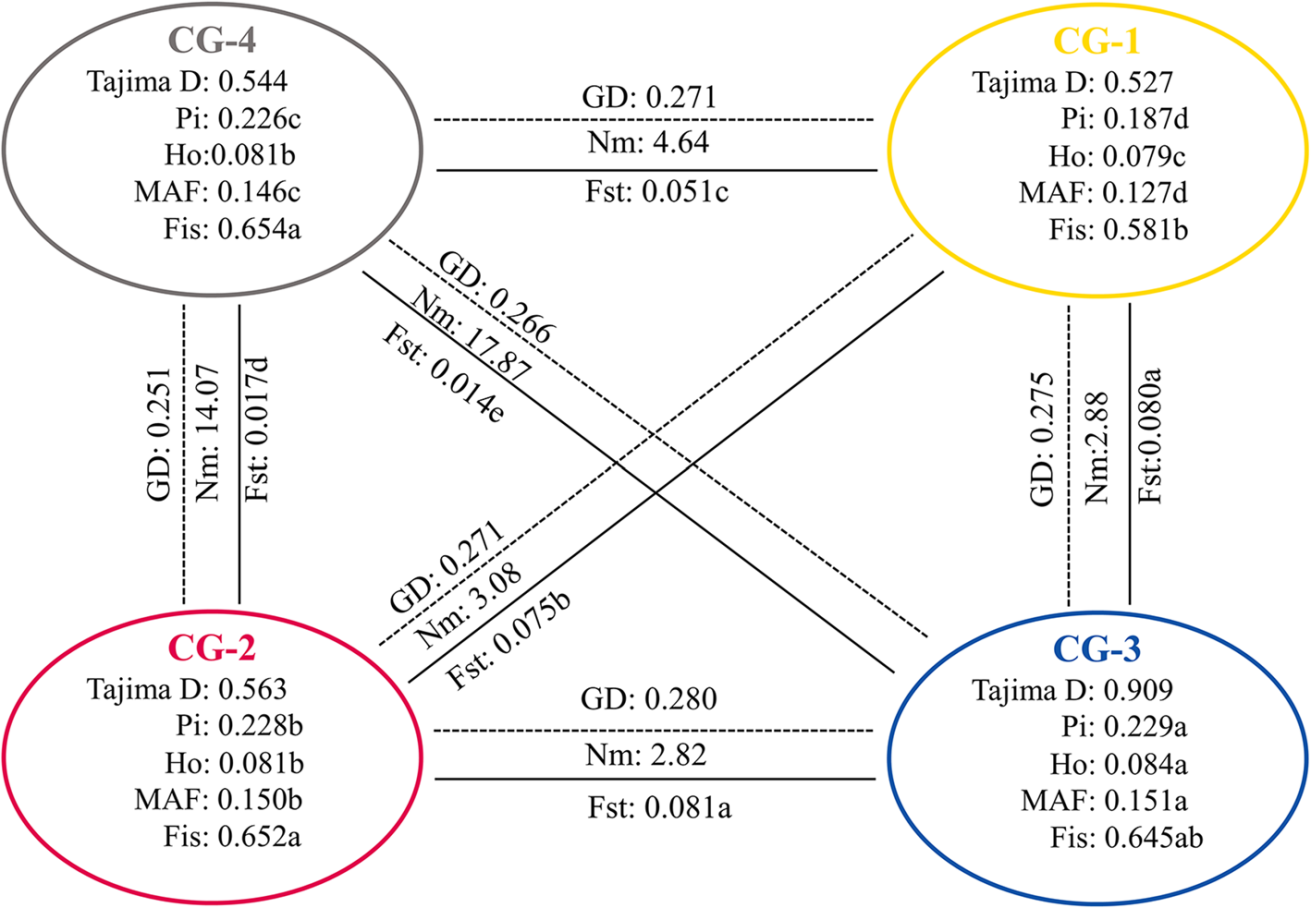
Genetic diversity of four inferred populations of 253 accessions. *Pi* nucleotide diversity, *Ho* observed heterozygosity, *MAF* minor allele frequency, *Fis* inbreeding coefficient, *GD* genetic distance, *Fst* differentiation coefficient, *Nm* gene flow. The different letters indicate a significant difference in *p* = 0.05 levels by the T-test. CG-1, CG-2 and CG-3 are pure groups and the CG-4 is the admixture group base on ADMIXTURE software at K = 3

Based on the population structure analysis, Tajima’s D, *Pi*, *Ho*, and *MAF* were calculated for CG-1, CG-2, CG-3, and CG-4 (Fig. 3). *Pi, Ho,* and *MAF* were significantly higher for CG-3 than for CG-1, CG-2, and CG-4. Moreover, *Pi, Ho*, and *MAF* were higher for CG-2 than for CG-1 and CG-4. However, *Ho* did not significantly differ between CG-2 and CG-4. *Pi, Ho,* and *MAF* were higher for CG-4 than for CG-1. All four groups had positive Tajima’s D values. Hence, they all underwent population bottlenecks, and/or balancing selection (Fig. 3).

Previous studies showed that *Fst* in the range of 0.00–0.05 indicate little divergence, while Fst in the range of 0.05–0.15 indicate moderate divergence [41–43]. We analyzed pairwise *Fst* across the four inferred groups. The mean *Fst* between CG-1 and CG-2, between CG-1 and CG-3, and between CG-2 and CG-3 were 0.075, 0.080, and 0.081, respectively. Thus, there is moderate divergence between CG-1 and CG-2, between CG-1 and CG-3, and between CG-2 and CG-3. The higher gene flow (*Nm*) was detected for CG-2 vs CG-4 and CG-3 vs CG-4, while the lowest *Nm* was detected for CG-2 vs CG-3. Therefore, there are more gene exchanges between CG-2 and CG-4, between CG-3 and CG-4 and less between CG-2 and CG-3. The highest GD was determined for CG-2 vs. CG-3 while the lowest GD was determined for CG-4 vs. CG-1. (Fig. 3).

### Core collection development

Core set was developed to select the minimum number of accessions representing the maximum diversity of the original population. This information can be used in molecular marker-assisted breeding, GWAS and other applications [45–47]. The maximum length subtree method implicated in DARwin v.6.0.17 was used to remove redundant accessions until the pruned edge and sphericity index percentage leveled off and corresponded to 195 accessions (Additional file 4: Fig. S1). These accessions were selected to represent the 253 cultivated-type tea accessions and are henceforth referred to as the ‘primary set’ (Additional file 1: Table S1). At the interval on the x-axis where the number of accessions decreased from 195 to 85, the pruned edge and sphericity index percentage gradually and stably increased. Therefore, the values did not significantly differ among 111 accessions and, the sphericity index and pruned edge had no significant impact after these accessions were removed (Additional file 4: Fig. S1). Eighty-five accessions were selected to represent all 253 cultivated-type tea accessions and are henceforth referred to as the ‘core set’. (Additional file 1: Table S1).

**Fig. 4:**
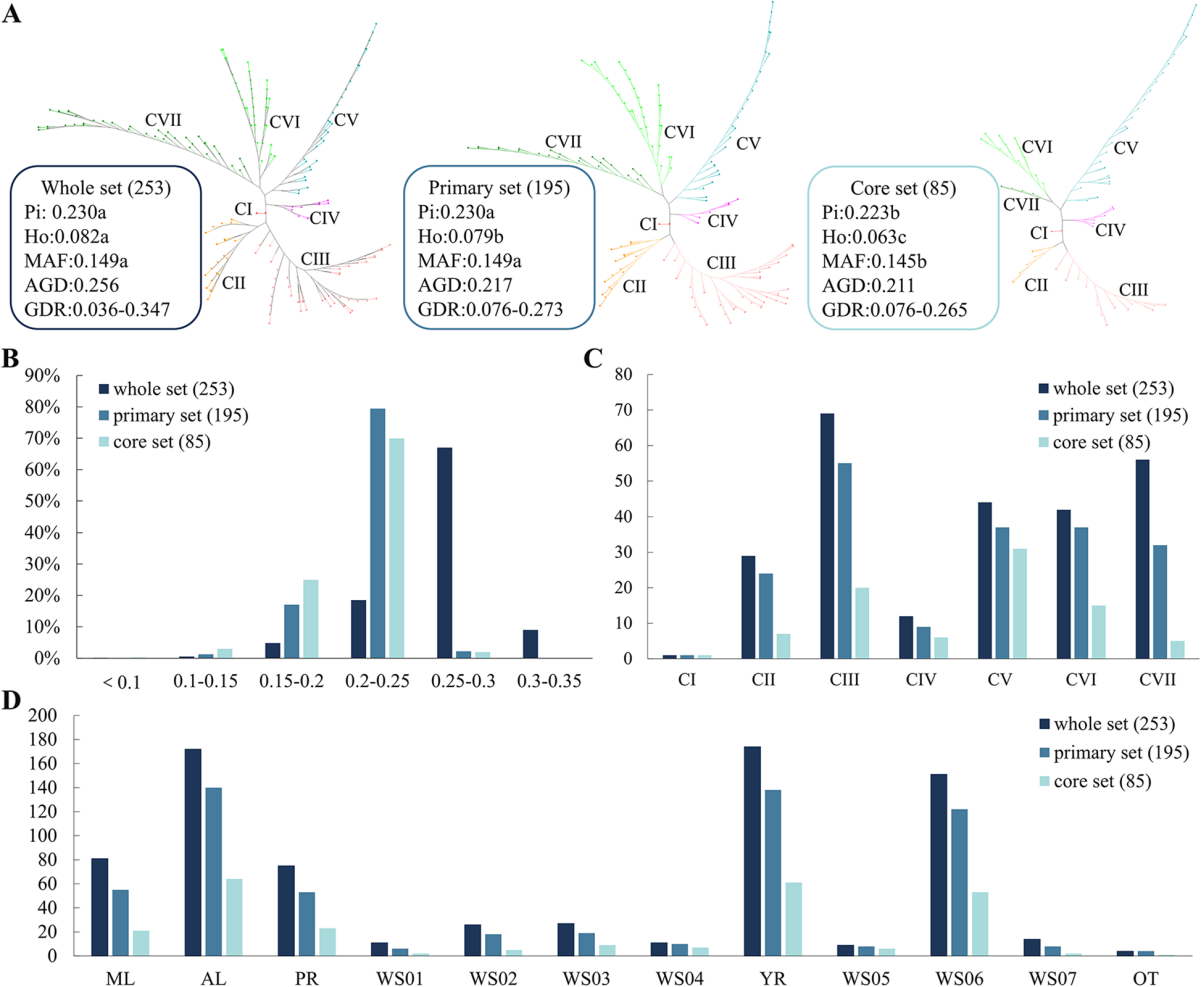
Summary of comparison information among primary, core and whole sets. **A** The NJ tree and genetic diversity of whole set, primary and core sets. *Pi* nucleotide diversity, *MAF* minor allele frequency, *AGD* average genetic distance, *GDR* genetic distance range. *CI* cluster I, *CII* cluster II, *CIII* cluster III, *CIV* cluster IV, *CV* cluster V, *CVI* cluster VI, *CVII* cluster VII. The different letters indicate a significant difference in *p* = 0.05 levels by the T-test; **B** Frequency distribution histogram of pairwise genetic distance of 253 accessions. **C** The histogram of the numbers of accessions of whole, primary and core sets in seven groups according to NJ tree. **D** The histogram of the numbers of accessions of whole set, primary set and core set in cultivated statuses (*ML* modern landraces, *AL* ancient landraces), two basins (*PR* the Pearl River Basin, *YR* Yangtze River Basin) and seven water systems (WS01 Liujiang River System, WS02 Hongshui River System, WS03 Beipanjiang River System, WS04 Nanpanjiang River System, WS05 Yuanjiang River System, WS06 Wujiang River System, WS07 Chishui River System; PR Pearl River Basin, YR Yangtze River Basin)

To estimate the quality of the core set and the primary set, we constructed the NJ tree and used the GD matrix to verify whether its backbone changed. Based on the NJ tree topology, the 253 cultivated-type tea plant accessions were divided into cluster I-VII (Fig. 4A). Cluster I contained one ancient landrace from WS02 of PR Basin. Cluster II consisted of 29 accessions including 15 modern and 14 ancient landraces that were distributed mainly in the YR Basin. Cluster III comprised 69 accessions including one modern and 68 ancient landraces distributed mainly in WS06. Cluster IV contained 12 accessions including one modern and 11 ancient landraces. Ten of the latter were from WS06, one modern landrace was from WS02, and one ancient landrace was from WS03. Cluster V consisted of 44 accessions of which 37 were ancient landraces and seven were modern landraces. There were 35 accessions from the YR Basin and nine from the PR Basin. Cluster VI comprised 42 accessions including 36 ancient and six modern landraces. There were 30 accessions from the PR Basin and 12 from the YR Basin. Cluster VII contained 56 accessions including 51 modern and five ancient landraces. There were 17 accessions from the PR Basin, 35 from the YR Basin, and four from OT (Additional file 1: Table S1, Fig. 4A).

We evaluated *MAF, Pi*, *Ho* and GD among whole set (253 cultivated-type tea accessions), primary set, and core set. The primary set had 100% of the whole set *Pi*, and *MAF*, 96.3% of the whole set *Ho*. The core set had 97% of the whole set *Pi* and *MAF*. *Ho* and GD slightly decreased for the core and primary sets. The minimum GD for the whole, primary and core sets were 0.036, 0.076 and 0.076, respectively (Fig. 4A, Table 4). The proportion of accessions with pairwise GD in the range of 0.200–0.250 had substantially increased for the primary and core sets (Fig. 4B). The foregoing results suggest that the core and primary sets contained accessions from all seven NJ tree clusters, both basins, all seven water systems, and both cultivation statuses. Thus, they represent the genetic diversity of the whole set (Fig. 4C, Fig. 4D, Additional file 1: Table S1).

**Table 4:** Genetic diversity of core, primary and whole sets of cultivated-type tea plant of Guizhou Plateau

group	simple size	Pi	Ho	MAF	AGD	GDR
core set	85	0.223b	0.063c	0.145b	0.211c	0.076–0.265
primary set	195	0.230a	0.079b	0.149a	0.217b	0.076–0.273
whole set	253	0.230a	0.082a	0.149a	0.265a	0.036–0.347

Note: *Pi* nucleotide diversity, *MAF* minor allele frequency, *AGD* average genetic distance, *GDR* Genetic distance range; In the same type and rows, The different letters indicate a significant difference in *p* = 0.05 levels by the T-test;

## Discussion

Previous studies have demonstrated that wind [33], water [34], animals [35, 36] and human activity [37] have influenced population distributions, the exchange of genetic information, the expansion of species, etc. However, the distribution characteristics and genetic diversity of cultivated-type tea plants are unclear. In present study, 253 cultivated-type tea accessions in the PR and YR Basins of Guizhou Plateau were collected for the first time. We analyzed their population structure, genetic diversity, core collection construction and mechanisms of genetic information exchange. Subsequent analyses revealed that the ancient hubs and basins played important roles in the distribution characteristics and genetic diversity of the cultivated-type tea plants in Guizhou Plateau.

### Genetic diversity of cultivated-type tea plants

GBS has been used to analyze the population structure of maize [48], common bean (*Phaseolus vulgaris L.*) [44], wheat [49], and tea [15, 50]. A previous study reported that 390.3 Gb clean data was obtained from 415 tea accessions and there was an average of 0.94 Gb clean data per accession. The researchers identified 1,001,372 initial and 79,016 high-quality SNPs [15]. By contrast, we generated 255.2 Gb clean data and an average of 1.00 Gb clean data per accession for 253 tea accessions. Our result showed that the high-quality SNPs accounted for 0.38% of the initial SNPs. That low than our previous study (8%) which mapping the sequence reads to fragment [15]. Indicated that the sequence data have relatively higher coverage and missing rate on the reference genome. While, under the same filter conditions, more high-quality SNPs were obtained in the present study than in the previous one [15]. Therefore, our sequence data could be used in subsequent analysis. Moreover, we obtained a transition/transversion ratio of 3.51, which was higher than those obtained for common bean (1.27) [44], apricot (1.78–1.79) [51], and lettuce (2.10) [52], but lower than that previously reported for tea (4.02) [15]. Thus, transitions better tolerated the natural resistance and might have consisted of synonymous mutations in protein-coding sequences [53].

*Pi*, *Ho* and *MAF* for the cultivated-type tea populations in the PR Basin were significantly higher than those for the cultivated-type tea populations in the YR Basin. *Fis* for the cultivated-type tea population in the PR Basin were lower than those for the cultivated-type tea population in the YR Basin (Table 2, Fig. 1C). Hence, the genetic diversity was significantly higher for the cultivated-type tea population in the PR Basin than for the cultivated-type tea population in the YR Basin. In the PR Basin, genetic diversity was relatively higher for WS02 and lower for WS04. In the YR Basin, genetic diversity was relatively higher for WS06 and lower for WS07 (Table 2). A plausible explanation for these results is that WS02 admixed modern landraces based on the initial ancient landraces and frequent genetic exchange occurred among individuals in both landraces. Wujiang River is a vital traffic route running through the entire Wujiang water system (WS06) and has promoted frequent genetic exchange among cultivated-type tea plant populations. WS04 and WS07 are located at the edges of the PR and YR basins and few corridors are available there to promote genetic exchange. All genetic diversity parameters except *Ho* were higher in the ancient than the modern landraces. Ancient landraces may not have been cultivated for breeding purposes [15]. Thus, for tea production purposes, modern landraces have been subjected to a certain degree of selection [15, 54].

Previous studies demonstrated that positive Tajima’s D values indicated population bottlenecks and/or balancing selection [39, 40]. Positive Tajima’s D values were observed in all populations in the present study. Hence, all of them may have been characterized by population bottlenecks and/or balancing selection (Table 2). *Fst* has been widely used as a measure of population structure. *Fst* in the range of 0.00–0.05 indicate little divergence while *Fst* in the range of 0 .05–0.15 indicate moderate divergence [41–43]. Pairwise *Fst* for all seven water systems were in the range of 0.00–0.05. Therefore, there were little divergence in these water systems.

### Population structure, PCA and phylogenetic tree analysis of cultivated-type tea plants

ADMIXTURE has been widely used to analyze the population structures of bean, pearl millet, and bread wheat [44, 55, 56]. The *k* value corresponding to the minimum CV error was deemed the optimal parameter to determine population structure. Here, we used the ADMIXTURE to analyze the population structure of cultivated-type tea plant and verified accuracy of its results via PCA and the NJ tree. ADMIXTURE categorized the 253 cultivated-type tea accessions in Guizhou Plateau into three pure groups (CG-1, CG-2, and CG-3) and one admixture group (CG-4) (Fig. 2A). PCA and NJ tree generated the same population structure output as ADMIXTURE.

Most of the accessions in CG-2 and CG-3 were ancient landraces from the PR and YR Basins, respectively. Moreover, most of the accessions in CG-2 and CG-3 were very remote from major rivers and ancient hubs (Fig. 1A, Additional file 1: Table. S3). We observed the highest GD and *Fst* and the lowest *Nm* between CG-2 and CG-3 (Fig. 3). Thus, there was little genetic communication between CG-2 and CG-3 and they assumed divergent evolutionary directions because of the physical distance between the YR and PR Basins. They are highly adaptable to the climates of the PR and YR Basins and are resistant to cold, drought, insects, and disease. They could serve as parental lines in hybridization or as germplasms in molecular breeding. CG-4 contained 25 modern and 34 ancient landraces. Most accessions from CG-4 were distributed near ancient hubs, while those were not near the ancient hubs were near the major rivers. (Additional file 1: Table. S3, Additional file 5: Fig. S1). *Fst* and GD were lower between CG-4 and CG-3 and between CG-4 and CG-2 than those between CG-4 and CG-1. *Nm* was higher between CG-4 and CG-3 and between CG-4 and CG-2 than those between CG-4 and CG-1. Hence, there was more genetic communication between CG-4 and CG-3 and between CG-4 and CG-2 than there was between CG-4 and CG-1. CG-1 represented modern landraces from tea garden and breeding varieties that are randomly distributed in Guizhou Plateau (Fig. 1A, Additional file 1: Table S1). CG-4 may have been the product of gene exchange between CG-2 and CG-3 via ancient hubs and major rivers and could have formed earlier than CG-1. Certain CG-4 accessions were used as a fence or for soil and water conservation while the others were planted in tea gardens for production tea. The latter may have gradually spread in response to road construction and the development of the modern tea industry, and evolved into CG-1. Hence, CG-4 accessions may have a complex evolutionary event and the utilization direction of CG-4 still needs further exploring. Here, CG-1 and CG-4 had lower genetic diversity than CG-3 and CG-2. Thus, CG-1 and CG-4 underwent artificial selection which spread because of cultivation. LD decay was slower for CG-1 than the other groups. Hence, CG-1 underwent strong artificial selection. They harbor the excellent traits required for tea production and could serve as germplasms in the selection of superior varieties nowadays.

### Core collection development

Tea germplasms are invaluable fundamental resources in biotechnology research and variety improvement. They have accelerated the development of tea plant genomics, genetics, and breeding [57–61]. Here, the core set was used to detect novel variations, select superior varieties, and furnish optimal germplasms because it consists of relatively smaller populations with comparatively higher genetic diversity [62]. Core collection development has been applied for cowpea [63], alpine plum [64], walnut [65], tea [50, 57] and other plants. However, no core collection has yet been developed for the cultivated-type tea accessions in Guizhou Plateau. In this study, we developed both the core and primary sets containing the samples from modern and ancient landraces, the YR and PR Basin, and the WS01–WS07 water systems. The proportions of accessions were consistent with the genetic diversity of the modern and ancient landraces. The primary and core sets included 77.0 and 33.6% of all individuals in the initial set, respectively. The primary set *Pi* and *MAF* were the same as those of the initial set. However, the core set *Pi*, *MAF*, and *Ho* were significantly lower than those of the primary set and initial set. Therefore, the primary set was selected as the population to carry out the GWAS while the core set was selected as the core population for multiple treatment settings analysis [46].

## Conclusions

We clustered 253 tea accessions into four populations including modern landraces (CG-1), PR Basin ancient landraces (CG-2), YR Basin ancient landraces (CG-3) and ancient hubs evolutionary (CG-4) groups. The genetic diversity of the YR ancient landraces group was higher than that of the PR ancient landraces group. The PR and YR ancient landraces groups went in different evolutionary directions because the PR and YR Basins physically diverged. The major rivers and ancient hubs were the main contributors to the genetic exchange between the PR and YR ancient landraces groups as well as the formation of the ancient hubs evolutionary group. We developed core and primary sets of cultivated-type tea plants, and the information therein can facilitate future tea germplasm protection and management, GWAS, and breeding.

## Methods

### Plant materials

A total of 253 cultivated-type tea plant accessions were collected and used in the present study [15]. Based on the research of Niu et al. [15], samples older than 100 years are referred to as “ancient landraces,” while samples from tea gardens are referred to as “modern landraces”. The 253 samples comprised 172 ancient and 81 modern landraces. Base on the position of Miaoling Mountain and the distribution of the major rivers and their tributaries, Guizhou Plateau was divided into the PR and YR Basins (Additional file 6: Fig. S1) and eight water systems (Additional file 6: Fig. S2) [17]. The PR Basin contained the Liujiang WS01, Hongshui WS02, Beipanjiang WS03 and Nanpanjiang WS04 water systems, while the YR Basin contained the Yuanjiang WS05, Wujiang WS06, Chishui WS07 and Niulan&Hengjiang WS08 water systems (Additional file 6: Fig. S2) [17]. Among the 253 tea accessions, four were introduced from Fujian, Zhejiang and Hunan Provinces, and became the main varieties cultivated in most tea gardens in Guizhou Plateau. The other 249 tea accessions were collected from 32 regions and distributed in the PY and YR Basins (Additional file 6: Fig. S1). Seventy-five tea accessions were distributed in the PR Basin and consisted of 22 modern and 53 ancient landraces. Of these, eleven were from WS01, 26 were from WS02, 27 were from WS03, and 11 were from WS04. There were 174 tea accessions in the YR Basin of which 55 were modern landraces and 119 were ancient landraces. Of the 174 tea accessions, nine were from WS05, 151were from WS06, and 14 were from WS07 (Additional file 1: Table S1).

### DNA extraction, library construction, and sequencing

A Plant Genomic DNA Rapid Extraction Kit (Beijing Biomed Gene Technology Co. Ltd., Beijing, China) was used to extract genomic DNA according to the manufacturer’s instructions. The DNA isolated from each sample was digested by the restriction endonucleases SacI and MseI (5 U; New England Biolabs (NEB), Ipswich, USA). The adaptors “SacAD and MseAD” had unique barcodes and were ligated with the DNA fragments. Separation was performed on 2% agarose gel; 500–550 bp long fragments were selected for amplification and sequenced on an Illumina Hi-Seq platform (Illumina, San Diego, CA, USA). The original paired-end sequence length was 150 bp [15, 66].

### Sequence alignment and SNP identification

The barcodes were used to de-multiplex the raw DNA reads and the adaptors were trimmed with a customized Perl script. Only reads with quality value > 5 were retained. They mapped to the reference genome (http://tpia.teaplant.org/) using BWA-MEM v. 0.7.10 (https://sourceforge.net/projects/bio-bwa/files/) with its default parameters [1]. The SNPs were filtered according to the methods of Niu et al. [15] according to several criteria. (1) The variants were bi-allelic SNPs. (2) “QUAL < 50.0 || QD < 2.0 || FS > 60.0 || MQ < 40.0 || Mapping Quality Rank Sum < -12.5 || Read Pos Rank Sum < -8.0” were used in GATK v. 3.7.0 (https://github.com/broadinstitute/gatk/releases) to filter the SNPs [38]. (3) SNPs with MAF > 0.05 or missing data rates < 20% were conserved with VCFtools v. 0.1.160 (https://github.com/vcftools/vcftools) [67]. The SNP density plot was drawn in CMplot v. 3.7.0 (https://rdrr.io/cran/CMplot/) [44]. A total of 112,072 SNPs from the 253 tea accessions were selected and subjected to the subsequent analysis (Additional files 7 and 8).

### LD and population structure

LD was calculated based on the correlation coefficient (r^2^) statistics for genome-wide unpruned pairwise SNPs using PopLDdecay v. 3.29 (https://github.com/BGI-shenzhen/PopLDdecay) with its default parameters [68].

VCFtools v. 0.1.160 was used to convert the VCF files into pedigree files [67]. ADMIXTURE v. 1.30 (http://dalexander.github.io/admixture/download.html) was used to estimate the proportions of admixtures among the cultivated-type tea populations by assuming that the number of ancestries (*k*) was in the range of 1–9. The optimal *k* value was confirmed based on the minimum CV error estimated by ADMIXTURE [69]. The threshold of the membership coefficient was set to 0.6 to distinguish between the pure and admixture groups [44]. PCA was performed in TASSEL v. 5.2.72 (https://tassel.bitbucket.io) [70]. An NJ tree was constructed in MEGA v. 10.2.4 (https://www.megasoftware.net/dload_win_gui) using its default parameter [71].

### Genetic diversity

*Ho*, MAF and *Fis* of each inferred population were calculated using Plink v. 1.90 (https://www.cog-genomics.org/plink2/) [72]. *Pi* and Tajima’s D of each inferred population and *Fst* of the pairwise inferred populations were computed using VCFtools [67]. *Nm* was calculated using formula *Nm* = (1-Fst) / 4Fst [73]. MEGA v. 10.2.4 was used to compute GD for the pairwise inferred populations. Significant differences between these indices were identified in SPSS v. 25 (IBM Corp., Armonk, NY, USA) [74].

### Core collection development

The NJ tree was generated based on the 112,072 SNPs. The ‘maximum length subtree’ function was used to develop the core collection for tea as previously described. The threshold and development steps of the core collections were fully described in a previous report [50].

## Supplementary Information

**Additional file 1: Table S1.** Information of 253 cultivated-type tea accessions used in the present study. Including the accession name, accession/clone/collection, the cultivation status, notes, source, basin, water system, the population structure-based grouping (Qi≥0.6) at K = 3, the cluster division base on NJ tree of development core collection, whether near ancient hubs and whether near major rivers. **Table S2.** Statistics of the number and ratio of the accessions of modern and ancient landraces, and both basins in four inferred populations. **Table S3.** Statistics of the number and ratio of the accessions of both basins, seven water systems, near ancient hubs and near major rivers in four inferred populations.

**Additional file 2: Table S1.** The quality control data of 253 cultivated-type tea accessions. **Table S2.** Statistics of Heterozygosity Rate of 112,072 SNPs in 253 cultivated-type tea accessions.

**Additional file 3: Figure S1.** Graph for CV error in the range of *k* = 1–9 of 253 cultivated-type tea accessions.

**Additional file 4: Figure S1.** The curve graph of the Sphericity Index percentage and pruned edge of 253 cultivated-type tea accessions.

**Additional file 5: Figure S1.** Diagram associating geographical distribution of Ming dynasty ancient routes/hubs [17, 76] and geographical distribution of the accessions in the ancient hubs evolutionary group (CG-4). Geographic distribution of each accession was represented by its’ pie chart of membership coefficient in ADMIXTURE on the Guizhou map. For the three membership coefficients, CG-1 (modern landraces group) was in yellow, CG-2 (PR ancient landraces group) was in red and CG-3 (YR ancient landraces group) was in blue in the pie chart.

**Additional file 6: Figure S1.** Geographic distribution map of tea accessions collection both basins analyzed in this study. (A) Geographical position. (B) Distribution map of both basins in Guizhou Plateau [17]. **Figure S2.** Geographic distribution map of tea accessions collection water systems analyzed in this study. (A) Geographical position. (B) Distribution map of eight water systems in Guizhou Plateau [17].

**Additional file 7.** Genotyping of 112,072 SNPs based on GBS in 125 cultivated-type tea accessions.

**Additional file 8.** Genotyping of 112,072 SNPs based on GBS in 128 cultivated-type tea accessions.

## Data Availability

The plant materials were growing in our resource nursery which are available from the corresponding author on reasonable request. The raw sequence data reported in this study have been deposited in the Genome Sequence Archive [75] in BIG Data Center, Beijing Institute of Genomics (BIG), Chinese Academy of Sciences, under accession number CRA001438 that is publicly accessible at http://bigd.big.ac.cn/gsa.

## Abbreviations

AGD: Average genetic distance
CV error: Cross-validation error
Fis: Inbreeding coefficient
Fst: Differentiation coefficients
GBS: Genotyping-by-sequencing
GD: Genetic distance
GDR: Genetic distance range
GWAS: Genome-wide association studies
Ho: Observed heterozygosity
LD: Linkage disequilibrium
MAF: Minor allele frequency
NJ tree: Neighbor-Joining tree
Nm: Gene flow
PCA: Principal component analyses
Pi: Nucleotide diversity
PR Basin: Pearl River Basin
SNPs: Nucleotide polymorphisms
YR Basin: Yangtze River Basin
