# Supplementary figures and images for: Population structure analysis to explore genetic diversity and geographical distribution characteristics of cultivated-type tea plant in Guizhou Plateau
Source: BMC Plant Biol. 2022 Jan 27;22:55. doi: 10.1186/s12870-022-03438-7 (PMC8793275; doi:10.1186/s12870-022-03438-7)

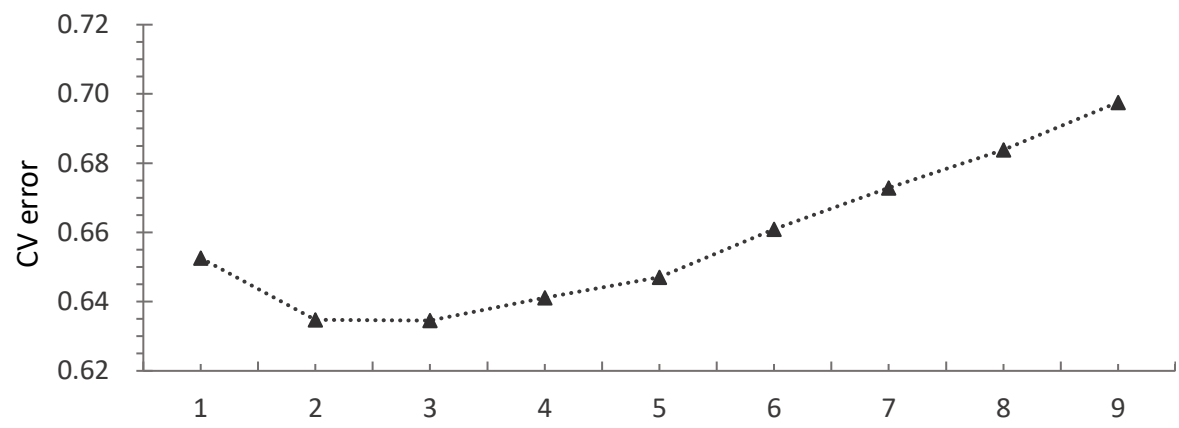

Supplement: Supplementary file 3 — Additional file 3: Figure S1. Graph for CV error in the range of k = 1–9 of 253 cultivated-type tea accessions. [file 12870_2022_3438_MOESM3_ESM.pdf]

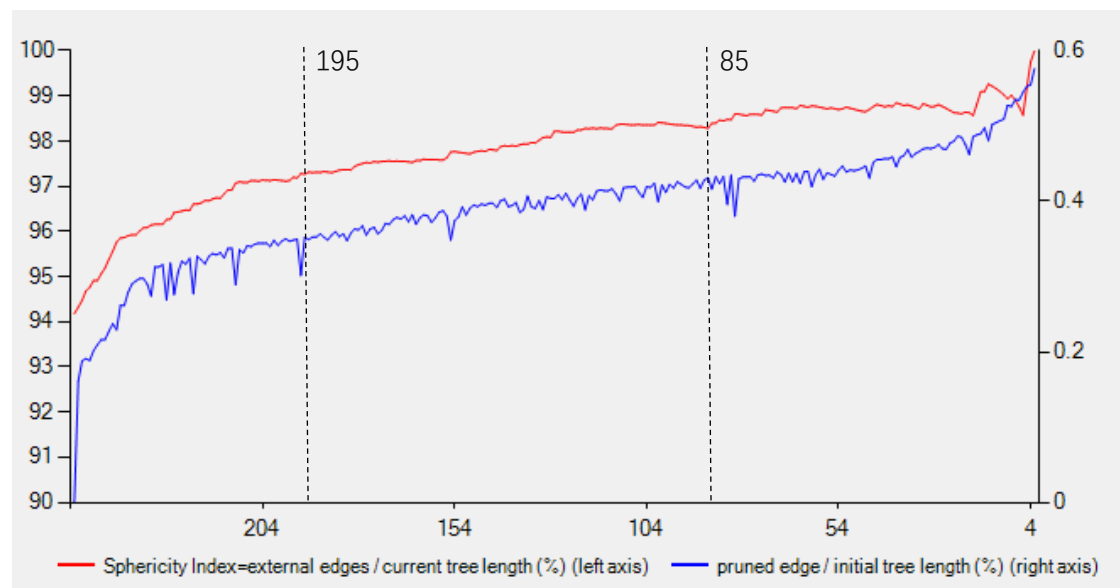

Supplement: Supplementary file 4 — Additional file 4: Figure S1. The curve graph of the Sphericity Index percentage and pruned edge of 253 cultivated-type tea accessions. [file 12870_2022_3438_MOESM4_ESM.pdf]

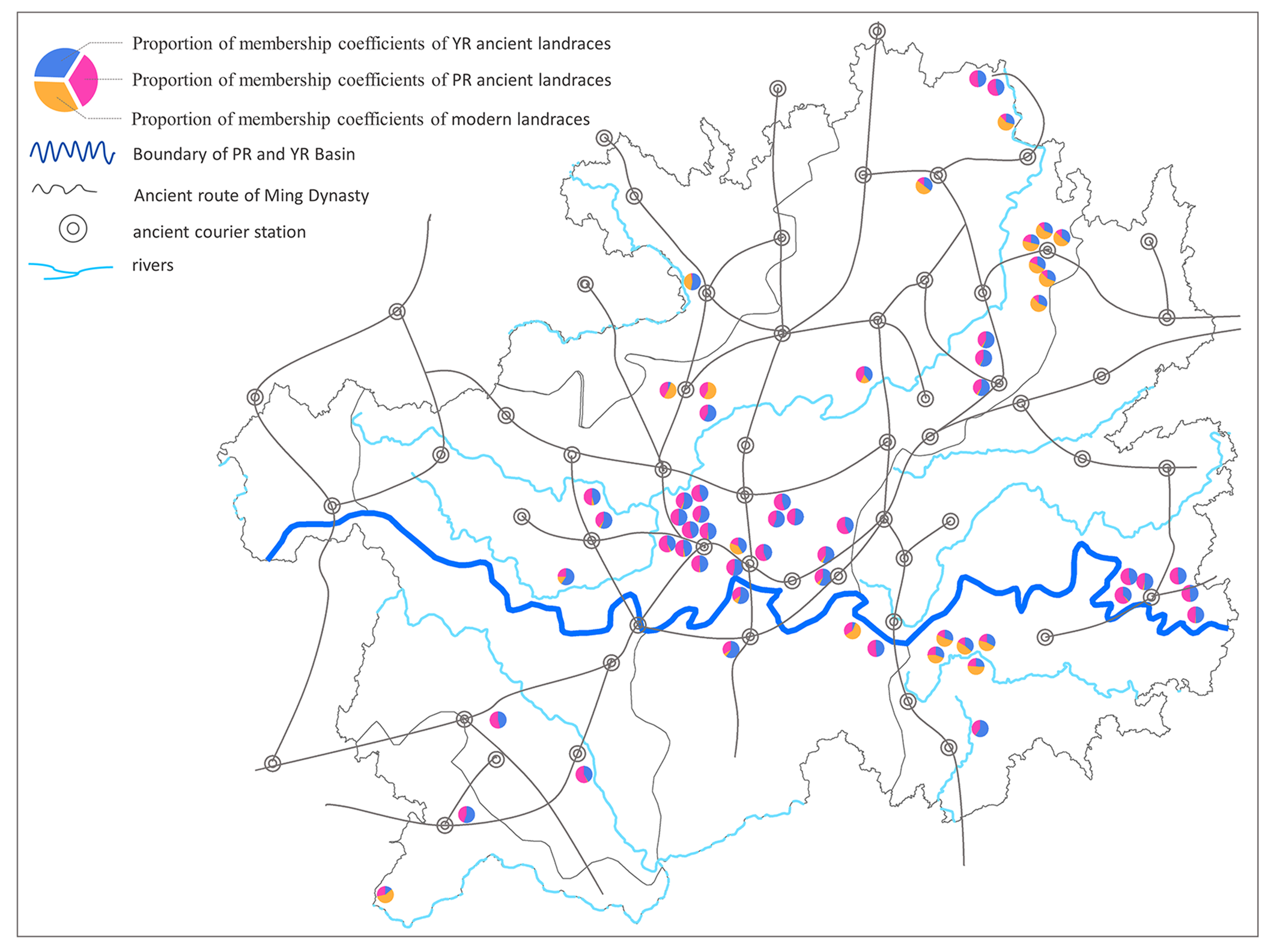

Supplement: Supplementary file 5 — Additional file 5: Figure S1. Diagram associating geographical distribution of Ming dynasty ancient routes/hubs [17, 76] and geographical distribution of the accessions in the ancient hubs evolutionary group (CG-4). Geographic distribution of each accession was represented by its’ pie chart of membership coefficient in ADMIXTURE on the Guizhou map. For the three membership coefficients, CG-1 (modern landraces group) was in yellow, CG-2 (PR ancient landraces group) was in red and CG-3 (YR ancient landraces group) was in blue in the pie chart. [file 12870_2022_3438_MOESM5_ESM.tif]
