# Supplementary material for: Population structure analysis to explore genetic diversity and geographical distribution characteristics of cultivated-type tea plant in Guizhou Plateau
Source: BMC Plant Biol. 2022 Jan 27;22:55. doi: 10.1186/s12870-022-03438-7 (PMC8793275; doi:10.1186/s12870-022-03438-7)
Supplement: Supplementary file 6 — Additional file 6: Figure S1. Geographic distribution map of tea accessions collection both basins analyzed in this study. (A) Geographical position. (B) Distribution map of both basins in Guizhou Plateau [17]. Figure S2. Geographic distribution map of tea accessions collection water systems analyzed in this study. (A) Geographical position. (B) Distribution map of eight water systems in Guizhou Plateau [17]. [file 12870_2022_3438_MOESM6_ESM.pdf]

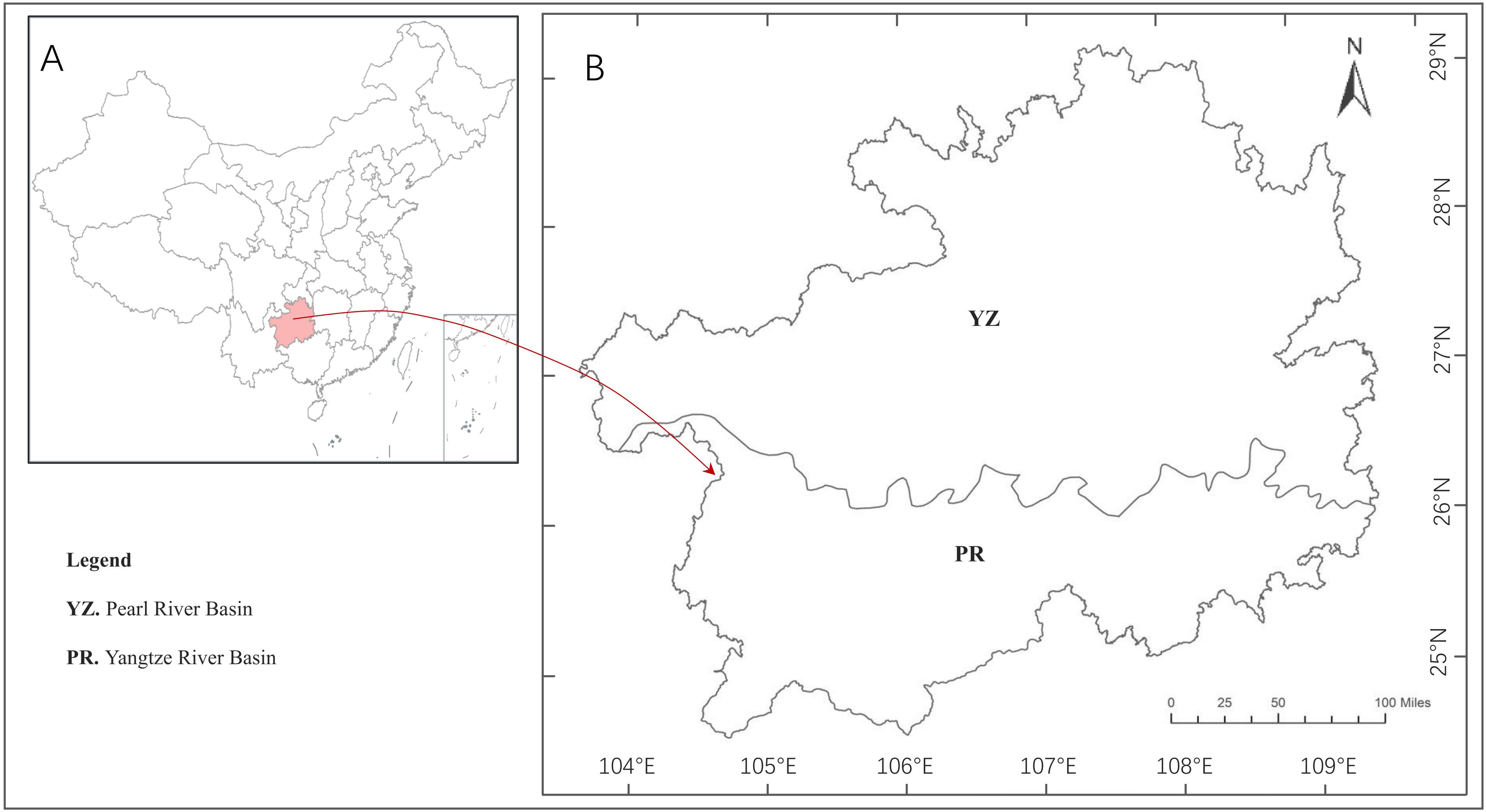
A
B
N
YZ
PR
Legend
YZ. Pearl River Basin
PR. Yangtze River Basin
0 25 50 100 Miles
104°E
105°E
106°E
107°E
108°E
109°E
25°N
26°N
27°N
28°N
29°N

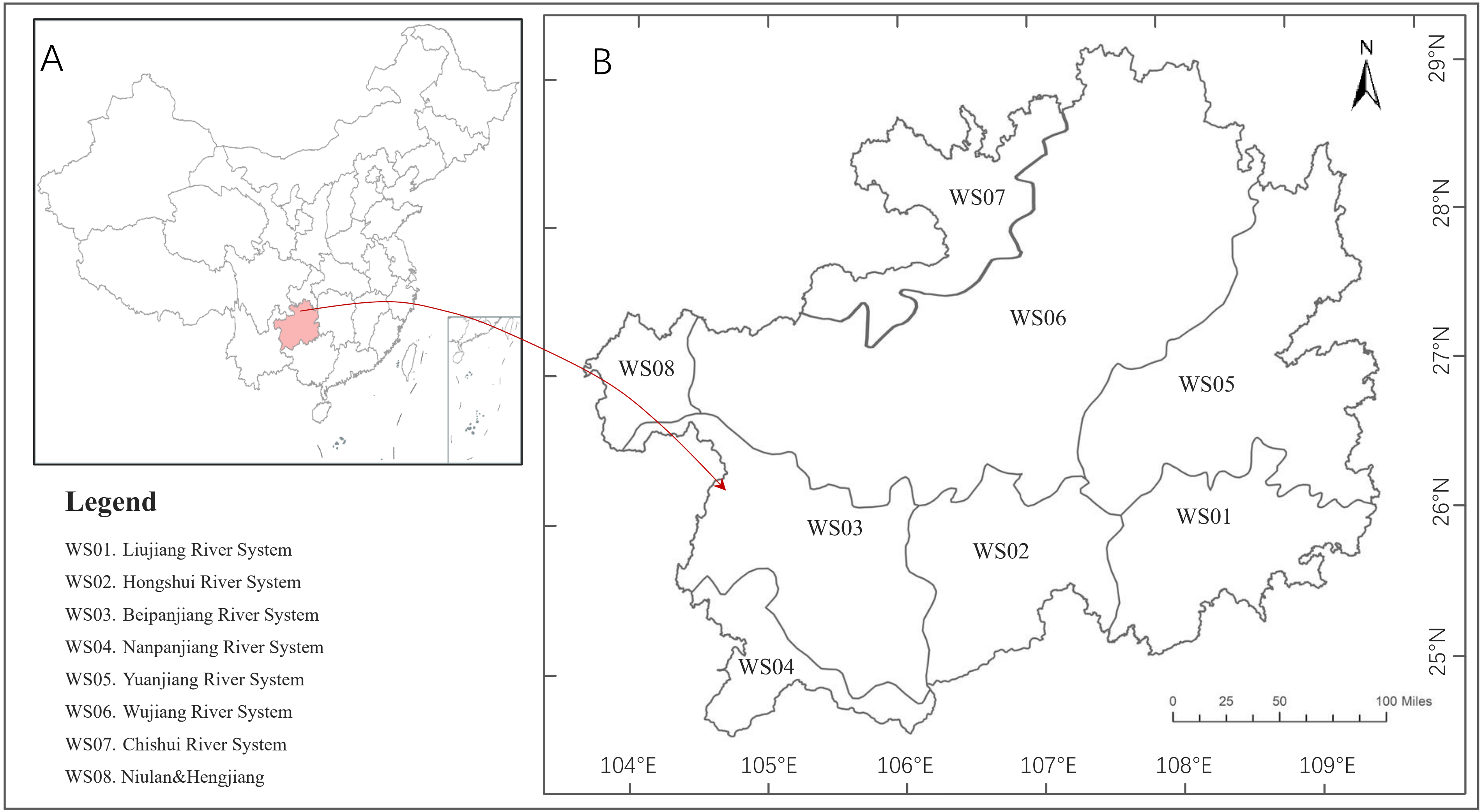
A
B
N
WS07
WS06
WS08
WS05
WS03
WS02
WS01
WS04
0
25
50
100 Miles
104°E
105°E
106°E
107°E
108°E
109°E
29°N
28°N
27°N
26°N
25°N
Legend
WS01. Liujiang River System
WS02. Hongshui River System
WS03. Beipanjiang River System
WS04. Nanpanjiang River System
WS05. Yuanjiang River System
WS06. Wujiang River System
WS07. Chishui River System
WS08. Niulan&Hengjiang
